# Wisdom from the past

**Published:** 1971-04

**Authors:** 


### Wisdom From The Past

**Extracts from this Journal 1896 and 1921**

### SEVENTY-FIVE YEARS AGO

**Book Review — Archives of Clinical Skiagraphy.** By SYDNEY ROWLAND. Vol. I., Part I. May, 1896. London: The Rebman Publishing Company Limited. —

It is characteristic of the present generation that an invention or discovery which half a century ago would have required years for its full development is nowadays often matured in as many months. Of this we have recently had a striking example in the "New Photography". Although it is only a few months since Röntgen announced his discovery of the X rays and their property of penetrating more or less completely substances opaque to ordinary light, the application of this property to the photography of the bones and other dense portions of the human body has already passed its first purely experimental stage, and is coming to be a recognised aid to medical diagnosis. Mr. Sydney Rowland, who is well known to readers of the *British Medical Journal* as an able exponent of the new art (named by him Skiagraphy), has just published the first number of the *Archives of Clinical Skiagraphy*. This he describes as "a series of collotype illustrations with descriptive text, illustrating applications of the New Photography to medicine and surgery". It contains excellent skiagrams of the body of a child aged three months (showing that the intestines, heart and liver cast shadows as well as the bones); of an arm with syphilitic disease of the radius; of a hand with needle in one of the fingers; and of the hand and knee-joint of a girl suffering from multiple exostoses. In connection with the latter, Mr. Rowland points out that the diseased bones are usually transparent to the X rays. The same thing was noticed in a similar case recently photographed at University College, Bristol. So marked was the effect, that after several attempts had been made a normal knee was photographed for comparison before it was realised that the bone and not the apparatus was the cause of the faintness of the bone-shadow. It is, we think, a pity that Mr. Rowland had painted out the tissues before printing his negative of the knee-joint, as the absence of contrast between them and the bones is thereby missed. The practice of touching up negatives taken for scientific purposes always tends to shake one's faith in their accuracy. The printing and paper of the Archives are all that can be desired, and the reproductions of the skiagrams clear and good. A neat portfolio is provided for containing the numbers.

— Vol. 14, p.186 (1896)

### FIFTY YEARS AGO

**New Building of the Bristol Homœopathic Hospital**

The foundation stone of the new hospital was laid by H.R.H. The Prince of Wales on June 10th. Through the generosity of the President, Mr. Melville Wills, Cotham House, standing in its spacious and beautifully-laid-out grounds of several acres, has been adapted and equipped as a temporary Hospital accommodating twenty-five patients. But when the new buildings are completed the existing house will be devoted to administration and the accommodation of the nursing staff, and 52 beds for patients will be provided in the new Hospital. The cost of providing the house and grounds and of the new building is a gift from the President in memory of his son, the late Capt. Bruce Melville Wills, who was killed in a brave attempt to bring in a wounded comrade early in the late war. It is intended that this new Hospital shall be as up to date and as perfectly equipped as possible, and we earnestly hope that the work carried on will be of much benefit to the patients and prove a worthy memorial of the gallant Captain Wills's self-sacrifice and devotion to his country's service.

— Vol. 38, p.125 (1921)

